# Biological activity of secondary metabolites of actinomycetes and their potential sources as antineoplastic drugs: a review

**DOI:** 10.3389/fmicb.2025.1550516

**Published:** 2025-05-08

**Authors:** Sun Rui, Guo Fengrui, Zhang Yining, Shao Hong, Yang Xuewen, Wang Changping, Yang Chunjia

**Affiliations:** ^1^College of Biology and Agriculture, Jiamusi University, Jiamusi, China; ^2^School of Basic Medicine, Jiamusi University, Jiamusi, China

**Keywords:** actinomycetes, antitumor, cancer, secondary metabolite, activity

## Abstract

Actinomycetes are an important group of Gram-positive bacteria, renowned for their ability to produce a wide array of structurally diverse and biologically active secondary metabolites. These secondary metabolites have significant applications in fields such as antimicrobial and antifungal treatments and show tremendous potential in cancer research. To comprehensively review the antitumor potential of actinomycetes-derived secondary metabolites, we conducted a systematic literature search across PubMed, Web of Science, and Scopus databases, covering the period from January 2019 to January 2024. The search used keywords including “actinomycetes,” “secondary metabolites,” “antitumor,” “cancer therapy,” “bioactivity,” and “clinical application.” A total of 95 relevant articles were identified through database searches. After applying inclusion and exclusion criteria, 87 articles were deemed eligible and fully reviewed in this article. These studies highlighted diverse structural classes of actinomycetes-derived antitumor compounds, including polyketides, non-ribosomal peptides, alkaloids, and terpenoids. Many of these metabolites exhibit potent anticancer properties through mechanisms such as inducing apoptosis, inhibiting proliferation, disrupting tumor microenvironment, and targeting key oncogenic signaling pathways. This review underscores the crucial role of actinomycetes secondary metabolites as an invaluable resource for antitumor drug discovery, offering new scientific insights into natural product-based cancer therapies, expanding the molecular toolbox for clinical oncology, and ultimately contributing to public health by advancing effective and innovative treatment options for cancer patients.

## Graphical abstract

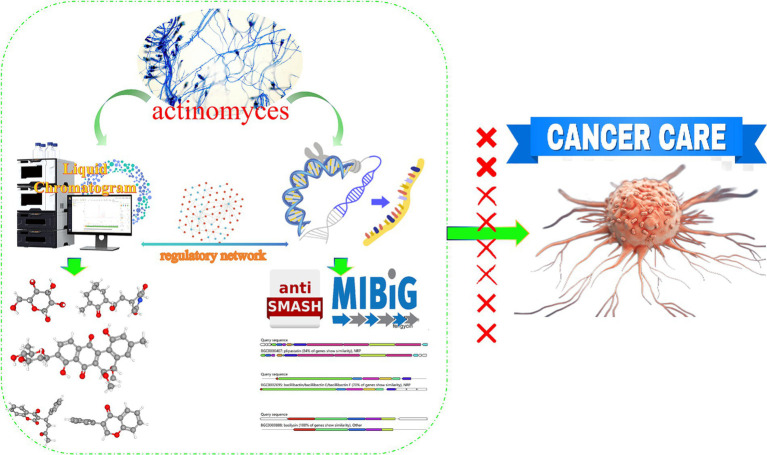

## Introduction

The phylum Actinomycetes is one of the most diverse groups among *Gram-positive bacteria*, comprising various genera, including Nocardia and Streptomyces. These bacteria are widely distributed in different natural environments, such as soil, lakes, caves, and oceans, demonstrating high diversity and ecological adaptability (Farda et al., 2022). Actinomycetes are extensively studied due to their unique morphological characteristics and physiological functions. They are typically characterized by branched mycelium and spores. Besides being the primary decomposers of organic matter in soil, actinomycetes play a crucial role in nutrient cycling within ecosystems (Devi et al., 2022).

Actinomycetes are also renowned for their ability to produce a wide range of secondary metabolites, which possess diverse biological activities such as antibiotics, antitumor agents, immunosuppressants, and enzyme inhibitors. Streptomyces alone produce approximately 100,000 antibiotic compounds, accounting for 70–80% of all naturally occurring bioactive products with pharmacological or agricultural applications (Alam et al., 2022). Consequently, actinomycetes have extensive applications in biotechnology and medicine. For example, streptomycin was the first effective antibiotic used to treat tuberculosis and is still widely used in clinical practice today.

Secondary metabolites are chemical compounds produced by organisms during their secondary metabolism. Unlike primary metabolites, such as sugars, fats, and proteins, which are essential for growth, development, or reproduction, secondary metabolites are not directly necessary for these basic processes. Instead, these compounds are produced under specific environmental conditions and possess particular biological activities or ecological functions, playing crucial roles in ecological adaptation and competition (Kolackova et al., 2023). They are significant for the survival and ecological fitness of organisms.

Secondary metabolites are widespread in nature and include antibiotics, toxins, pigments, growth regulators, and aromatic compounds. They have important applications in medicine, agriculture, and the food industry due to their various biological activities, such as antimicrobial, antiviral, anticancer, and antioxidant properties. For example, the discovery and application of antibiotics have greatly advanced modern medicine. Flavonoids, a type of plant secondary metabolite, are extensively studied for their antioxidant and anticancer potential (Al-Khayri et al., 2023; Elshafie et al., 2023).

Actinomycetes, particularly the genus *Streptomyces*, have been proven to be a source of various clinically used antitumor drugs, such as doxorubicin and actinomycin D. The secondary metabolites of actinomycetes exert antitumor effects through multiple mechanisms, including inducing apoptosis, inhibiting angiogenesis, and blocking tumor cell proliferation. With advancements in genomics and metabolomics, a vast number of unexplored biosynthetic gene clusters have been identified in actinomycetes, representing potential sources of novel antitumor compounds. The issues of drug resistance and side effects associated with traditional antitumor drugs have become increasingly prominent. Therefore, developing structurally novel secondary metabolites from actinomycetes with unique mechanisms of action offers promising new therapeutic options. In recent years, global investment in the research and development of new antitumor drugs has continued to grow, and actinomycete-derived secondary metabolites, due to their distinctive biological activities, have become a key focus in new drug development.

Actinomycetes-derived secondary metabolites have long been recognized for their diverse biological activities, including significant anticancer properties. Several well-known compounds, such as doxorubicin, actinomycin D, and mitomycin C, have demonstrated potent antitumor effects by inducing apoptosis, inhibiting angiogenesis, and modulating key oncogenic pathways. Over the past decades, advances in genomics and metabolomics have revealed a vast number of unexplored biosynthetic gene clusters (BGCs) in actinomycetes, suggesting that many novel bioactive compounds remain undiscovered. However, despite these findings, the full therapeutic potential of actinomycetes remains underutilized.

Many BGCs remain silent under conventional laboratory conditions, requiring innovative activation strategies such as the OSMAC approach, genome mining, and co-cultivation techniques to unlock their metabolic potential. Additionally, challenges persist in the field, including low production yields, difficulties in compound purification, and the frequent rediscovery of known metabolites, which hinder the development of new therapeutic agents. Recent studies have increasingly focused on marine-derived actinomycetes as a promising source of structurally unique and biologically active compounds with anticancer potential.

Given these advancements and challenges, this review provides a comprehensive synthesis of recent findings on actinomycetes-derived secondary metabolites with antitumor activity. It highlights their chemical diversity, mechanisms of action, and emerging strategies for discovery and optimization, offering insights into their potential applications in cancer therapy.

To comprehensively review the antitumor potential of actinomycetes-derived secondary metabolites, we conducted a systematic literature search across three major scientific databases: PubMed, Web of Science, and Scopus. The search period covered publications from January 2019 to January 2024. Search terms included combinations of keywords such as “actinomycetes,” “secondary metabolites,” “antitumor,” “cancer therapy,” “bioactivity,” and “clinical application.”

Studies were included if they focused on: the isolation of secondary metabolites from actinomycetes. The structural characterization of these metabolites. The evaluation of their antitumor activity, and the investigation of their mechanisms of action.

The following studies were excluded: conference abstracts, non-peer-reviewed reports, and publications in languages other than English.

Through this systematic search process, a total of 95 articles were initially identified. After applying inclusion and exclusion criteria, 87 articles were deemed eligible and fully reviewed. These selected studies comprehensively covered diverse structural classes of actinomycetes-derived antitumor compounds, including polyketides, non-ribosomal peptides, alkaloids, and terpenoids. The reviewed articles not only highlighted the structural diversity but also provided insights into their mechanisms, such as inducing apoptosis, inhibiting proliferation, modulating tumor microenvironments, and targeting key oncogenic signaling pathways.

## Main biological activities of actinomycetes secondary metabolites

The secondary metabolites of actinomycetes have been widely used in biomedicine and agriculture, showing rich biological activities due to their diverse chemical structures and unique mechanisms of action. It mainly includes the following aspects:

### Antitumor activity

Many secondary metabolites from actinomycetes have been found to possess antitumor activities, inhibiting the growth and proliferation of tumor cells through various mechanisms. Actinomycin D, produced by the genus Streptomyces, is a well-known antitumor antibiotic (Adeluola et al., 2022) and was the first drug approved for clinical cancer treatment (Plummer and Walker, 1958). It functions by binding to DNA and inhibiting RNA synthesis. By intercalating between DNA base pairs, it prevents DNA-dependent RNA polymerase from transcribing mRNA. This action inhibits protein synthesis and leads to cell death, making it effective against rapidly dividing cancer cells.

Mitomycin C, an antibiotic produced by *Streptomyces caespitosus*, is primarily used in cancer treatment and to prevent scarring after glaucoma surgery. It exhibits its antitumor activity through strong DNA cross-linking. Upon reductive activation, it forms mitosene, which cross-links DNA by N-alkylating two DNA bases, thus hindering DNA replication and transcription and ultimately inhibiting the growth and division of cancer cells (Moynahan et al., 2001). Additionally, drugs like avermectin, derived from actinomycetes, also exhibit antitumor activity.

This article reviews the antitumor components discovered from actinomycetes secondary metabolites in recent years, providing valuable references for the development and clinical application of new antitumor drugs.

### Antimicrobial activity

Since the “Golden Age of Antibiotics,” spanning 1940 to 1960, actinomycetes have been recognized as a rich source of various secondary metabolites. A substantial number of bioactive natural products have been identified from microbial sources, particularly in the field of antimicrobial drug discovery. Numerous widely-used antibiotics have been derived from actinomycetes (Ngamcharungchit et al., 2023a).

Oxytetracycline is an important antibiotic closely associated with actinomycetes. Discovered in the 1940s by American microbiologist named Albert Schatz and his team from soil-dwelling Streptomyces discovered that oxytetracycline inhibits bacterial protein synthesis, making it effective against a wide range of bacterial infections (Cheng et al., 2022; Chopra and Roberts, 2001). Since its discovery, it has been widely used in clinical settings to treat respiratory, urinary, intestinal, and skin infections.

Streptomycin, another antibiotic derived from Streptomyces, specifically *Streptomyces griseus*, is effective against a variety of *Gram-positive* and *Gram-negative bacteria*, especially *Mycobacterium tuberculosis* (Kohanski et al., 2010). Its discovery in the 1940s significantly reduced tuberculosis mortality rates, marking a major breakthrough in medical history.

Additionally, erythromycin, obtained from *Streptomyces erythreus*, belongs to the macrolide class of antibiotics and has broad-spectrum antibacterial activity. It has played a crucial role in treating respiratory infections, skin infections, and some sexually transmitted diseases (Kanoh and Rubin, 2010).

The significance of actinomycetes in antibiotic discovery is undeniable, as they are the source of many key antibiotics, including streptomycin, erythromycin, and tetracycline. Modern genomics and metabolic engineering technologies have further enhanced the research and application of actinomycetes secondary metabolites, ensuring these microorganisms continue to play a vital role in developing new antibiotics.

### Other biological activities

Beyond their antitumor and antimicrobial activities, secondary metabolites from actinomycetes exhibit various other biological activities, including antiparasitic, antioxidant, antihyperglycemic, and anti-inflammatory properties.

Avermectin, in addition to its anticancer activity, possesses broad-spectrum insecticidal and antiparasitic properties. It works by interfering with chloride ion channels in nerve and muscle cells, leading to paralysis and the death of the parasites. This mechanism makes it effective against a wide range of parasites and pests. Ivermectin, a derivative of avermectin, is particularly effective in treating and preventing various parasitic infections, such as onchocerciasis (river blindness) and lymphatic filariasis (Ngamcharungchit et al., 2023a).

Rapamycin, an immunosuppressant produced by Streptomyces, is widely used in immunosuppressive therapy following organ transplantation. It exerts potent immunosuppressive activity by inhibiting the mTOR (mammalian target of rapamycin) signaling pathway. mTOR is a key regulator of cell growth, proliferation, and survival. By inhibiting mTOR, rapamycin can prevent the proliferation of T cells and B cells, thereby suppressing the immune response (Liu and Sabatini, 2020).

The diversity and complexity of secondary metabolites from actinomycetes provide a rich resource of natural products for medical research and demonstrate significant potential in the development of new drugs and the treatment of various diseases.

## Antitumor components in secondary metabolites from actinomycetes

Cancer is one of the deadliest diseases worldwide, claiming nearly 10 million lives in 2020 (Sung et al., 2021). This dire statistic underscores the urgent need for medical research to develop new anticancer compounds to reduce the incidence and mortality rates globally. Researchers are exploring natural sources, particularly active substances from marine resources, in search of new anticancer drugs with fewer side effects (Ngamcharungchit et al., 2023a). Secondary metabolites derived from actinomycetes represent a crucial reservoir of potential anticancer drugs. For example, Streptomyces species among actinomycetes produce numerous anticancer agents, including doxorubicin, actinomycin, and bleomycin.

**Figure 1:**
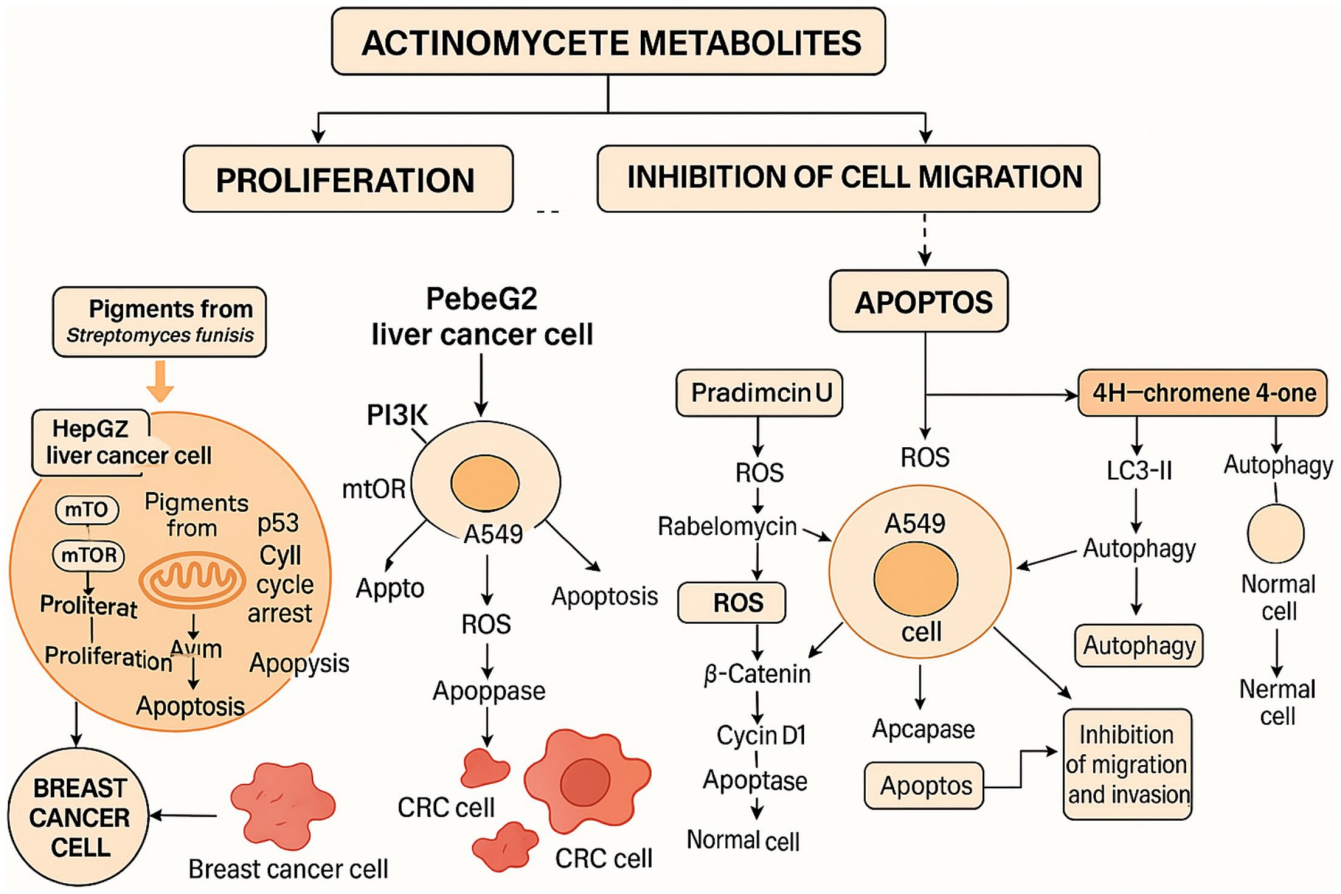
Actinomycete-derived metabolites modulate cancer cell signaling pathways.

We have reviewed secondary metabolites from actinomycetes with anticancer potential published between 2019 and 2024. It is important to note that many studies have only explored the cytotoxicity of these metabolites on tumor cells without examining their effects on normal cells. Further research is needed to determine their applicability in the anticancer field. Additionally, some extracts of secondary metabolites exhibit promising antitumor activity; however, studies often assess these effects using mixtures of crude extracts (Ibrahim et al., 2023; Saini et al., 2024; Ribeiro et al., 2020; Salehghamari et al., 2022; Zamora-Quintero et al., 2022) rather than isolating the active components. Despite this, their initial screening experiments provide valuable data that warrant further investigation (Ribeiro et al., 2020; Almuhayawi et al., 2021) (Figure 1).

### Antitumor activity against breast cancer

**Figure 2:**
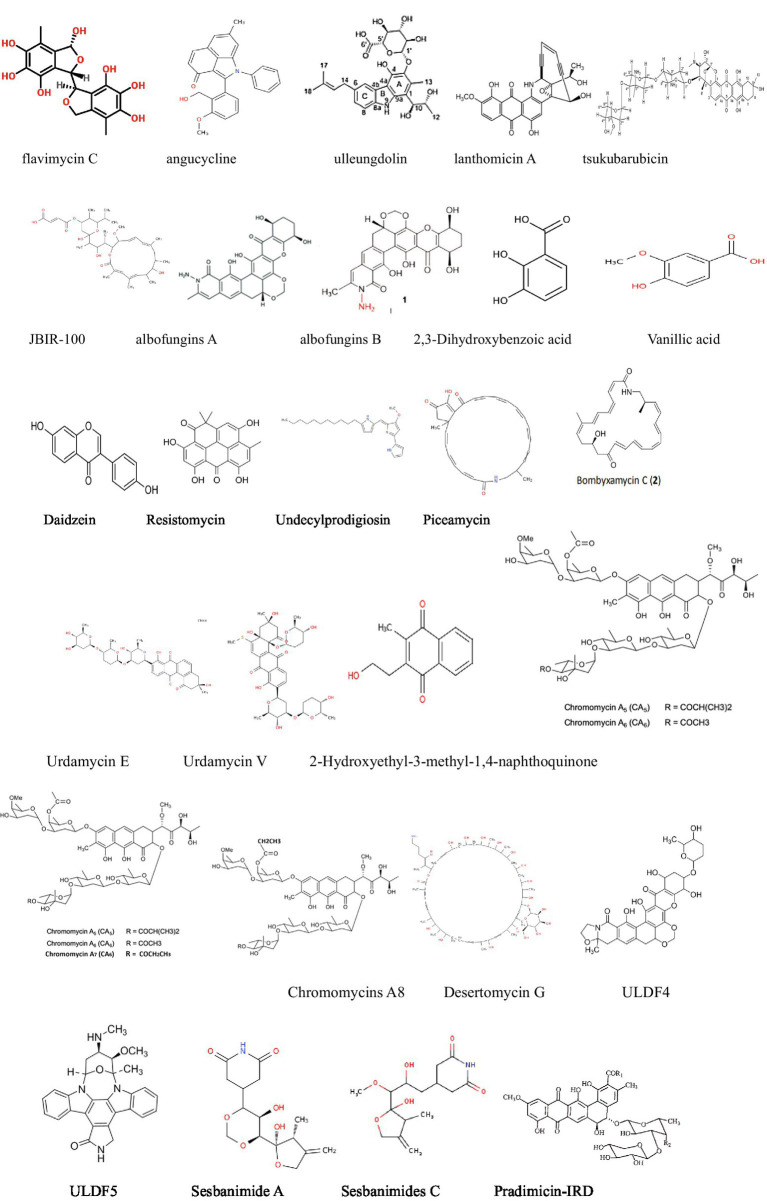
Compounds with antitumor activity against breast cancer from actinomycetes secondary metabolites.

Breast cancer is the most prevalent cancer among women worldwide, with approximately 2.3 million new cases in 2022, accounting for 11.6% of all cancer cases (Bray et al., 2024). It is also a leading cause of cancer-related deaths in women, especially in developed countries. Despite progress in existing treatments, significant gaps remain. Recent studies have found that certain secondary metabolites from actinomycetes exhibit antitumor activity against breast cancer, showing promise as potential new clinical drugs (Table 1 and Figure 2).

**Table 1:** Compounds with antitumor activity against breast cancer from actinomycetes secondary metabolites.

Name/Compounds	Organism	Cell line	Cytotoxicity	References
Flavimycin C	*Epicoccum nigrum* Ann-B-2	MCF-7	IC50: 2.7 μM	Elnaggar et al. (2024)
Angucycline/Angucyclinone derivatives of dehydroxyaquayamycin	*Rhizosphere soil Streptomyces* sp. SYP-A7185	MCF-7	IC50: 16.58 μM	Xue et al. (2023)
Ulleungdolin	*Streptomyces* sp. 13F051	MDA-MB-231	Cell migration was inhibited by approximately 42% at 10 μM and 100% at 25 μM	Hwang et al. (2022)
Lanthomicin A	*Streptomyces chattanoogensis* L10	MCF-7	IC50: 5.98 μM	Liu et al. (2022)
Tsukubarubicin	*Streptomyces tsukubaensis*	MDA-MB-231	IC50: 2.93 nM	Wu et al. (2021)
JBIR-100	*Streptomyces* sp.	MCF-7	IC50: 2.3 nM	Chiu et al. (2021)
JBIR-100	*Streptomyces* sp.	MDAMB-231	IC50: 5.7 nM	Chiu et al. (2021)
Albofungins A	*Streptomyces chrestomyceticus*	MCF-7	IC50: 0.005 μM	She et al. (2021)
Albofungins B	*Streptomyces chrestomyceticus*	MCF-7	IC50: 0.012 μM	She et al. (2021)
2,3-Dihydroxybenzoic acid	*Streptomonospora arabica* VSM-25	MDA-MB-231	IC50: 10 μg/mL	Magot et al. (2023)
Vanillic acid	*Streptomonospora arabica* VSM-25	MDA-MB-231	IC50: 1 μg/mL	Magot et al. (2023)
Daidzein	*Streptomonospora arabica* VSM-25	MDA-MB-231	IC50: 1 g/mL	Magot et al. (2023)
Vanillic acid	*Streptomonospora arabica* VSM-25	MCF-7	IC50: 10 g/mL	Magot et al. (2023)
Daidzein	*Streptomonospora arabica* VSM-25	MCF-7	IC50: 1 g/mL	Magot et al. (2023)
Resistomycin	*Streptomyces* sp. EGY34	HCT116	IC50: 19.8 μM	Elmallah et al. (2020)
Undecylprodigiosin	Actinomycete RA2	HCT116	IC50: 17.5 μM	Elmallah et al. (2020)
Piceamycin	*Streptomyces* sp. SD53 strain	MDA-MB-231	IC50: 0.66 μM	Shin et al. (2020)
Bombyxamycin C	*Streptomyces* sp. SD53 strain	MDA-MB-231	IC50: 1.00 μM	Shin et al. (2020)
Urdamycin E	*Streptomyces* sp. OA293	MCF-7	IC50: 4.92 μg/mL	Dan et al. (2020)
Urdamycin V	*Streptomyces* sp. OA293	MCF-7	IC50: 5.08 μg/mL	Dan et al. (2020)
2-Hydroxyethyl-3-methyl-1,4-naphthoquinone	*Actinoalloteichus cyanogriseus*	MDA-MB-435	IC50: 10.59 μM	Zhang X. et al. (2021)
Chromomycins A5	*Streptomyces* sp.	MCF-7	IC50: 3.7 μM	Florêncio et al. (2022)
Chromomycins A6	*Streptomyces* sp.	MCF-7	IC50: 14.7 μM	Florêncio et al. (2022)
Chromomycins A7	*Streptomyces* sp.	MCF-7	IC50: 133.0 μM	Florêncio et al. (2022)
Chromomycins A8	*Streptomyces* sp.	MCF-7	IC50: 10.5 μM	Florêncio et al. (2022)
Desertomycin G	*Streptomyces althioticus*	MCF-7	IC50: 3.8 μM	Braña et al. (2019)
ULDF4	*Streptomyces* sp.	MCF-7	IC50: 0.0139 mg/mL	Davies-Bolorunduro et al. (2019)
ULDF5	*Streptomyces* sp.	MCF-7	IC50: 2.176 mg/mL	Davies-Bolorunduro et al. (2019)
Sesbanimide A	*Stappia indica*	MDA-MB-23	GI50: 6.4 × 10^−07^	Kačar et al. (2021)
Sesbanimides C	*Labrenzia aggregata*	MDA-MB-23	GI50: 1.6 × 10^−07^–8.6 × 10^−09^	Kačar et al. (2021)
Pradimicin-IRD	*Amycolatopsis* sp. IRD-009	MCF-7	IC501.55 μM	Bauermeister et al. (2019)

For example, Xue et al. (2023) isolated and identified several anthraquinone metabolites, including six new compounds, from *Streptomyces* sp. SYP-A7185. Compounds 1–6 and 9 exhibited moderate to strong cytotoxicity against five human cancer cell lines (A549, HepG2, MCF-7, MDA-MD-231, and MGC-803). Molecular docking results indicated that these compounds inhibit cancer cell migration by targeting MMP2. Apart from that, another study isolated a novel secondary metabolite, ulleungdolin, during co-cultivation of *Streptomyces* sp. 13F051 and *L. minima*15S071. Bioactivity evaluation revealed its anti-invasive effects on breast cancer cells (Hwang et al., 2022). Additionally, 2-Hydroxyethyl-3-methyl-1,4-naphthoquinone, isolated from *Actinoalloteichus cyanogriseus*, showed cytotoxicity against MDA-MB-435 cells with an IC50 of 10.5 μM (Hwang et al., 2022).

ULDF 4 and ULDF 5, isolated from 12 different marine actinomycete strains named *Streptomyces bingchengensis* ULS 14, demonstrated cytotoxicity against human MCF-7 breast cancer cells with IC50 values of 0.0139 and 2.176 mg/mL (Xue et al., 2023), respectively. Coloraducin A5-A8, isolated from *Streptococcus* sp., reduced the viability of human MCF-7 tumor cells with IC50 values of 3.7, 14.7, 133.0, and 10.5 nM. Polyketide compounds sesbanimide A and sesbanimides C–F, produced by *Stappia indica* and *Labrenzia aggregata*, respectively, exhibited cytotoxicity against MDA-MB-231 cells with GI50 values ranging from 1.6 × 10^−07^ to 8.6 × 10^−09^ (Florêncio et al., 2022).

Moreover, Wu et al. (2021) successfully activated a silent gene cluster (TSU) and identified a novel bioactive anthracycline antibiotic, tsukubaricin, through overexpression of putative antibiotic regulatory proteins (SARPs) and bioactivity-guided screening in *Streptomyces griseus*. Tsukubaricin demonstrated superior antitumor activity against various human cancer cell lines, particularly breast cancer cell lines, compared to the clinically used doxorubicin.

### Antitumor activity against colorectal cancer

**Figure 3:**
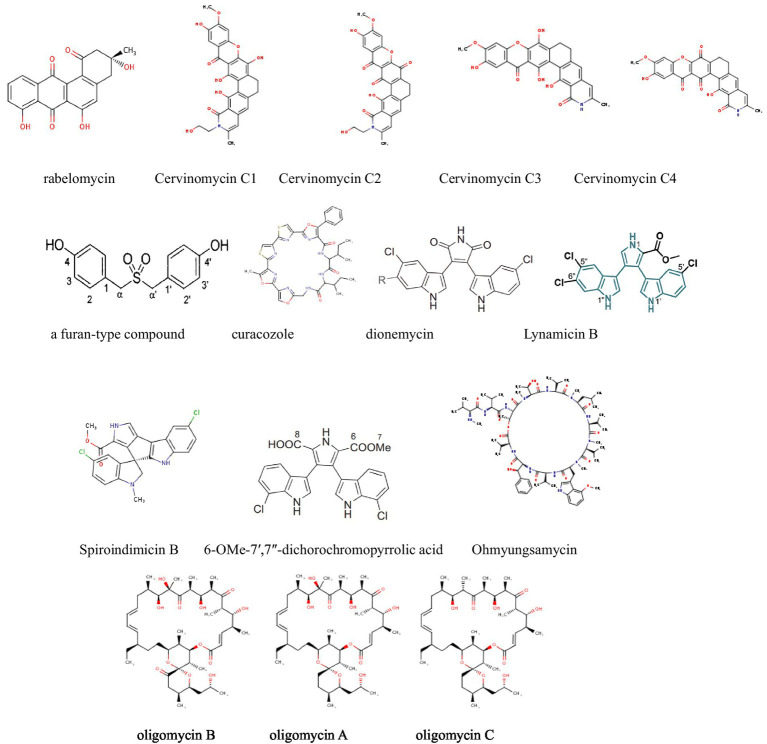
Compounds with antitumor activity against colorectal cancer from actinomycetes secondary metabolites.

Colorectal cancer (CRC) is the third most common cancer worldwide. According to global cancer statistics, approximately 1.9 million new cases of CRC were reported in 2022, accounting for 9.6% of all cancer cases, making it the second deadliest cancer globally (Chen et al., 2021). We have summarized the findings from recent research papers on the antitumor activity of secondary metabolites from actinomycetes against colorectal cancer (Table 2 and Figure 3).

**Table 2:** Compounds with antitumor activity against colorectal cancer from actinomycetes secondary metabolites.

Name/Compounds	Organism	Cell line	Cytotoxicity	References
Rabelomycin	SCSIO LCY30	SW480	IC50: 1.57 μM (SI 4.49)	Li Y. et al. (2023)
Tsukubaricin	*Streptomyces tsukubaensis*	HCT116	IC50: 24.0 nM	Wu et al. (2021)
Resistomycin	*Streptomyces* sp. EGY1 and EGY34	HCT116	IC50: 7.6 μM	Jagannathan et al. (2021)
Undecylprodigiosin	Actinomycetes strain RA2	HCT116	IC50: 21.34 μM	Elmallah et al. (2020)
Cervinomycin C1	*Streptomyces* sp. CPCC 204980	HCT116	IC50: 7.6 nM	Hu et al. (2020)
Cervinomycin C2	*Streptomyces* sp. CPCC 204980	HCT116	IC50: 80.2 nM	Hu et al. (2020)
Cervinomycin C3	*Streptomyces* sp. CPCC 204980	HCT116	IC50: 0.9 nM	Hu et al. (2020)
Cervinomycin C4	*Streptomyces* sp. CPCC 204980	HCT116	IC50: 1.1 nM	Hu et al. (2020)
Piceamycin	*Streptomyces* sp. SD53 strain	HCT116	IC50: 0.22 μM	Shin et al. (2020)
Bombyxamycin C	*Streptomyces* sp. SD53 strain	HCT116	IC50: 1.01 μM	Shin et al. (2020)
A furan-type compound	*Streptomyces* sp. VN1	HCT116	IC50: 123.7 μM	Nguyen et al. (2020)
Curacozole	*Streptomyces curacoi* mutant strain R25	HCT-116	IC50: 8.6 nM	Kaweewan et al. (2019)
Dionemycin	Marine *Streptomyces* sp. SCSIO 11791	HCT-116	IC50: 4.3 μM	Song et al. (2020)
Lynamicin B	Marine *Streptomyces* sp. SCSIO 11791	HCT-116	IC50 = 8.7 μM	Song et al. (2020)
Spiroindimicin B	Marine *Streptomyces* sp. SCSIO 11791	HCT-116	IC50 = 2.2 μM	Song et al. (2020)
6-OMe-7′,7″-dichorochromopyrrolic acid	Marine *Streptomyces* sp. SCSIO 11791	HCT-116	IC50: 13.1 μM	Song et al. (2020)
Ohmyungsamycin	*Streptomyces* strain SNJ042	HCT-116	IC50 = 7.61 μM	Byun et al. (2020)
Oligomycin B	*Streptomyces* sp. FXY-T5	DLD-1	IC50: 5.80 μM	Feng et al. (2024)
Oligomycin A	*Streptomyces* sp. FXY-T5	DLD-1	IC50: 9.55 μM	Feng et al. (2024)
Oligomycin C	*Streptomyces* sp. FXY-T5	DLD-1	IC50: 8.73 μM	Feng et al. (2024)
Pradimicin-IRD	*Amycolatopsis* sp. IRD-009	HCT-116	IC50: 0.8 μM	Bauermeister et al. (2019)

Feng et al. (2024) isolated 12 oligomycin compounds from the marine-derived *Streptomyces* sp. FXY-T5 and evaluated their antiproliferative effects on DLD-1 cells. Compounds with IC50 values less than 10 μM are reported in the table. In addition, Li Y. et al. (2023) employed the OSMAC method to isolate three cyclic peptide compounds from the marine-derived *Streptomyces SCSIO* LCY30. The results show that, they investigated the toxicity of these compounds on normal and tumor cells, finding that rabelomycin exhibited selective cytotoxicity against the colon cancer cell line SW480 (IC50 = 1.57 μM) compared to the normal intestinal epithelial cell line NCM460 (IC50 = 7.05 μM), with a selectivity index (SI) of 4.49, therefore, indicating its potential as an anticancer drug for colorectal cancer.

### Antitumor activity against liver cancer

**Figure 4:**
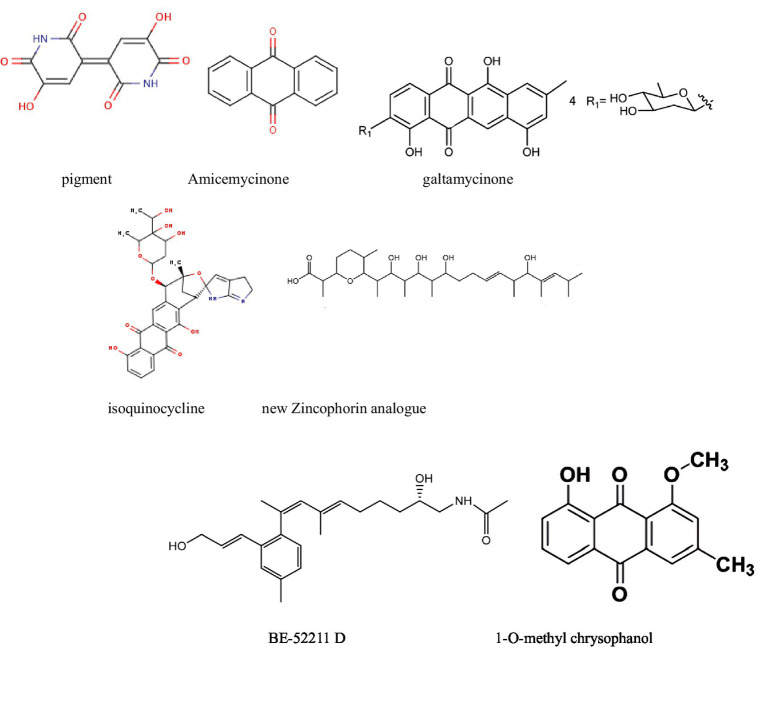
Compounds with antitumor activity against liver cancer from actinomycetes secondary metabolites.

Liver cancer is the sixth-most common cancer globally. According to global cancer statistics, approximately 900,000 new cases of liver cancer were reported in 2022, accounting for 4.7% of all cancer cases, making it the third deadliest cancer worldwide (Bray et al., 2024). Recent research papers on the antitumor activity of secondary metabolites from actinomycetes against liver cancer are summarized in Table 3 and Figure 4.

**Table 3:** Compounds with antitumor activity against liver cancer from actinomycetes secondary metabolites.

Name/Compounds	Organism	Cell line	Cytotoxicity	References
Pigment	*Streptomyces tunisiensis* W4MT573222	HepG-2	IC50: 2 mg/mL	Ibrahim et al. (2023)
Angucycline/Angucyclinone derivatives of dehydroxyaquayamycin	Rhizosphere soil *Streptomyces* sp. SYP-A7185	HepG-2	IC50: 12.18 μM	Xue et al. (2023)
Amicemycinone	Rhizosphere soil *Streptomyces* sp. SYP-A7185	HepG-2	IC50: 16.46 μM	Xue et al. (2023)
Galtamycinone	Rhizosphere soil *Streptomyces* sp. SYP-A7185	HepG-2	IC50: 20.83 μM	Xue et al. (2023)
Albofungins A	*Streptomyces chrestomyceticus*	HepG2	IC50: 0.02 μM	She et al. (2021)
Albofungins B	*Streptomyces chrestomyceticus*	HepG2	IC50: 0.33 μM	She et al. (2021)
Isoquinocycline	*Micromonospora* 28ISP2-46T	HepG2	IC50: 13.3 μM	Back et al. (2021)
Piceamycin	*Streptomyces* sp. SD53 strain	SK-HEP-1	IC50: 0.32 μM	Shin et al. (2020)
Bombyxamycin C	*Streptomyces* sp. SD53 strain	SK-HEP-1	IC50: 1.87 μM	Shin et al. (2020)
New zincophorin analogue	NEAU-wh-3-1	HepG2	IC50: 9.6 μg/mL	Wang et al. (2020)
BE-52211 D	NEAU-wh-3-1	HepG2	IC50: 10.83 μg/mL	Wang et al. (2020)
1-O-methyl chrysophanol	*Amycolatopsis thermoflava* SFMA-103	Lung cancer	IC50: 10.3 μM	Chandrasekhar et al. (2021)

For instance, pigments extracted from *Streptomyces tunisiensis* W4MT573222 showed IC50 values of 0.2 mg/mL (HepG-2), 1,114 μg/mL (A549), and 1,043 μg/mL (PAN1). The IC50 for Vero cells (normal cells) was 2014 μg/mL (2 mg/mL), indicating a high safety profile for the extracted pigments. Therefore, these findings suggest that the pigments demonstrate selective cytotoxicity against cancer cells while maintaining relative safety for normal cells. Furthermore, qualitative separation and development of the active substances in the pigments using methods such as mass spectrometry could, in turn, aid in the discovery of new anticancer drugs (Ibrahim et al., 2023).

### Antitumor activity against lung cancer

**Figure 5:**
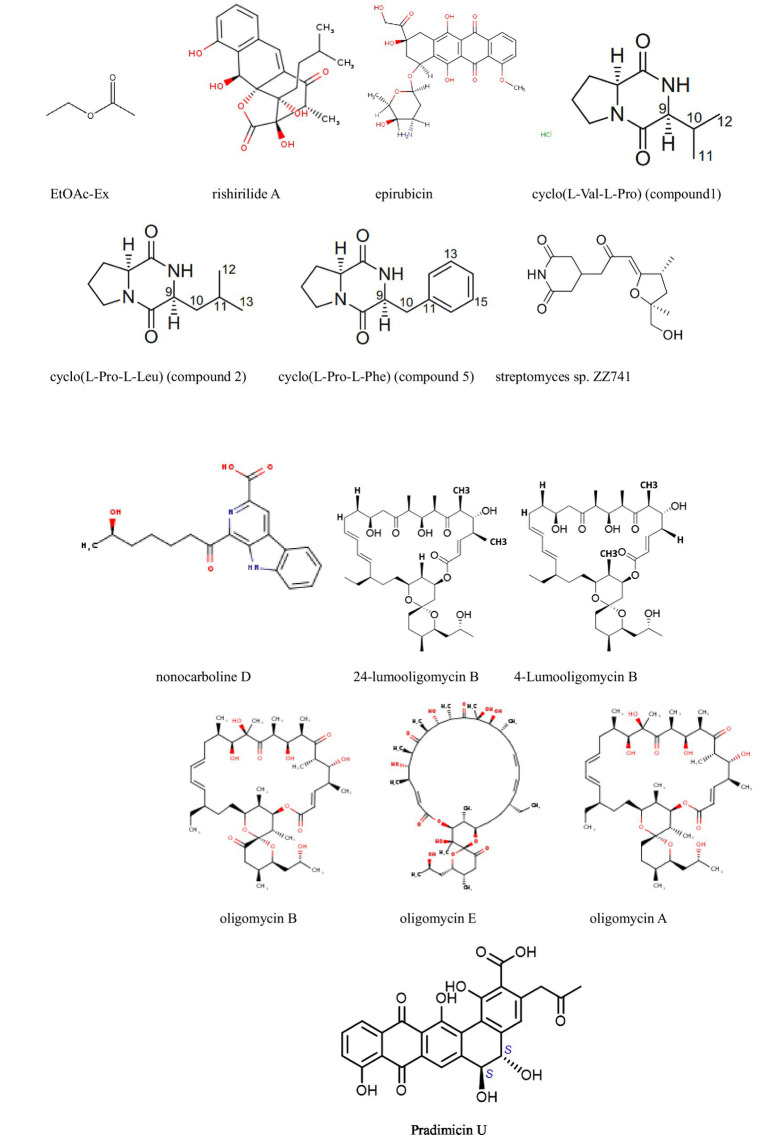
Compounds with antitumor activity against lung cancer from actinomycetes secondary metabolites.

Lung cancer has the highest incidence rate and is the second-leading cause of cancer-related deaths worldwide (Li C. et al., 2023). With the increasing resistance to existing anticancer drugs, we have reviewed recent secondary metabolites from actinomycetes with antitumor activity against lung cancer (Table 4 and Figure 5).

**Table 4:** Compounds with antitumor activity against lung cancer from actinomycetes secondary metabolites.

Name/Compounds	Organism	Cell line	Cytotoxicity	References
EtOAc-Ex	*Streptomyces* sp. KS20	A549	IC50: 94.73 μg/mL	Chakraborty et al. (2023)
Methanolic extract (the main component is C_17_H_29_NO_14_)	*Streptomyces sennicomposti* GMY01	HTB	IC50: 33.75 μg/mL	Widada et al. (2023)
Pigment	*Streptomyces tunisiensis* W4MT573222	A549	IC50: 1.1 mg/mL	Ibrahim et al. (2023)
Lupinacidin A	*S. xanthophaeus*	A549	IC50: 11.37 μM	Zhang et al. (2022)
Rishirilide A	*S. xanthophaeus*	A549	IC50: 24.7 μM	Zhang et al. (2022)
Epirubicin	*Streptomyces clavuligerus*	Hop-62	GI50: 409.54 μg/mL	Saini et al. (2024)
Angucycline/angucyclinone derivatives of dehydroxyaquayamycin	Rhizosphere soil *Streptomyces* sp. SYP-A7185	A549	IC50: 5.58 μM	Liu et al. (2022)
Amicemycinone	Rhizosphere soil *Streptomyces* sp. SYP-A7185	A549	IC50: 10.83 μM	Liu et al. (2022)
Galtamycinone	Rhizosphere soil *Streptomyces* sp. SYP-A7185	A549	IC50: 6.51 μM	Liu et al. (2022)
Lanthomicin A	*Streptomyces chattanoogensis* L10	A549	IC50: 0.17 μM	Liu et al. (2022)
Cyclo(L-Val-L-Pro) (compound1)	*Streptomyces griseorubens* f8	A549	The number of invaded and migrated cells was gradually decreased with increasing concentrations of compounds 1, 2, 3, and 5	Li X. et al. (2023)
Cyclo(L-Pro-L-Leu) (compound 2)	*Streptomyces griseorubens* f8	A549		Li X. et al. (2023)
Cyclo(L-Pro-L-Phe) (compound 5)	*Streptomyces griseorubens* f8	A549		Li X. et al. (2023)
Tsukubarubicin	*Streptomyces tsukubaensis*	A549	IC50: 7.41 nM	Wu et al. (2021)
*Streptomyces* sp. ZZ741	Streptoglutarimide H	A549, H157, H460, H1299, H1703, and PC9	IC50: 1.69–5.24 μM	Wu et al. (2021)
Piceamycin	*Streptomyces* sp. SD53 strain	A549	IC50: 0.28 μM	Shin et al. (2020)
Bombyxamycin C	*Streptomyces* sp. SD53 strain	A549	IC50: 0.78 μM	Shin et al. (2020)
Nonocarboline D	Rare actinobacterium *Nonomuraea* sp.	A549	IC50: 1.7 μM	Primahana et al. (2020)
A furan-type compound	*Streptomyces* sp. VN1	A549	IC50: 58.64 μM	Feng et al. (2024)
24-Lumooligomycin B	*Streptomyces* sp. FXY-T5	A549	IC50: 3.67 μM	Feng et al. (2024)
4-Lumooligomycin B	*Streptomyces* sp. FXY-T5	A549	IC50: 5.72 μM	Feng et al. (2024)
Oligomycin B	*Streptomyces* sp. FXY-T5	A549	IC50: 1.81 μM	Feng et al. (2024)
Oligomycin E	*Streptomyces* sp. FXY-T5	A549	IC50: 5.91 μM	Feng et al. (2024)
Oligomycin A	*Streptomyces* sp. FXY-T5	A549	IC50: 3.55 μM	Feng et al. (2024)
Pradimicin U	FMUSA5-5T	NCI-H187	IC50: 5.69 g/mL	Duangupama et al. (2024)

Duangupama et al. (2024) identified a new secondary metabolite, pradimicin U, from the actinomycete strain FMUSA5-5T. This dihydrobenzo[a]naphthacenequinone compound exhibited strong antitumor activity against human small cell lung cancer (NCI-H187) with an IC50 of 5.69 μg/mL, while showing weak toxicity against other tumors. Similarly, Bano et al. (2024) developed nano-silver particles coated with secondary metabolites. However, although the reference value is low due to the lack of product separation and the introduction of silver, these particles exhibited strong activity against the A549 cell line.

In addition, the methanol crude extract from *Streptomyces sennicomposti* GMY01 showed moderate bioactivity against HeLa (cervical cancer) and HTB (human lung cancer) cells *in vitro*, with IC50 values of 27.31 and 33.75 μg/mL, respectively. The main component was identified as C_17_H_29_NO_14_, with a minor component being mannotriose (C_18_H_32_O_16_, 1.96%). Molecular docking analysis indicated that mannotriose has an affinity for autophagy proteins (mTORC_1 and mTORC_2) in cancer cells (Widada et al., 2023).

Furthermore, Liu et al. (2022) identified a new pentangular polyphenolic compound, Lanthomicin A, by activating a cryptic aromatic polyketide BGC. This compound exhibited antiproliferative activity against the lung cancer cell line A-549 with an IC50 of 0.17 μM. Likewise, Li et al. isolated 12 oligomycin compounds and evaluated their antiproliferative effects on A549 cells, reporting five compounds that induced significant G1 phase cell cycle arrest through the β-catenin signaling pathway, significantly reducing Cyclin D1 and PCNA expression.

Finally, Yang et al. obtained five bioactive secondary metabolites from marine-derived *Streptomyces griseus* f8. Notably, compounds 1, 2, 3, and 5 were found to inhibit the invasion and migration of A549 cells (lung cancer cells) for the first time, thus demonstrating their potential as novel anticancer drugs (Li X. et al., 2023).

### Antitumor activity against prostate cancer

**Figure 6:**
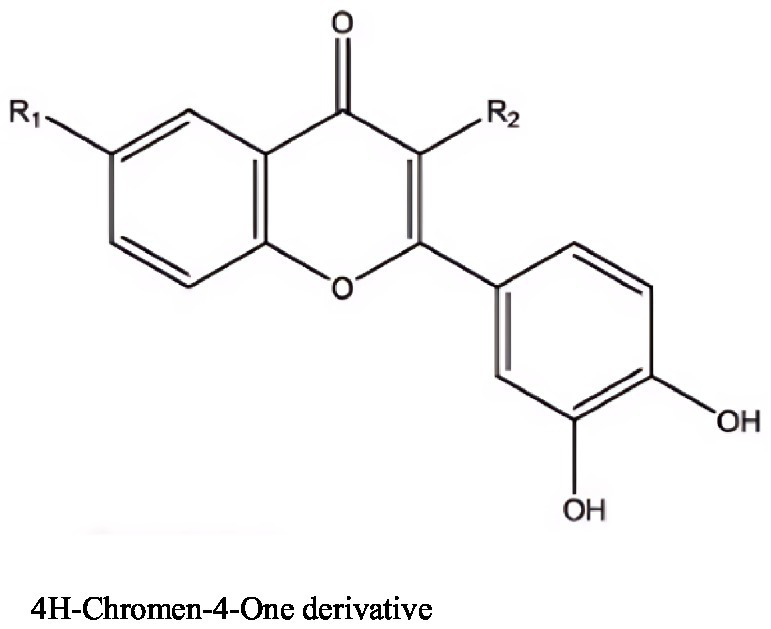
Compounds with antitumor activity against prostate cancer from actinomycetes secondary metabolites.

Prostate cancer is the second-most common cancer globally (Table 5 and Figure 6). According to global cancer statistics, approximately 1.5 million new cases of prostate cancer were reported in 2022, accounting for 7.9% of all cancer cases, making it the fifth deadliest cancer worldwide. In this context, Lin et al. (2021) isolated a secondary metabolite, Lu01-M62, from marine-derived *Streptomyces* sp. and studied its antiproliferative activity against prostate cancer cells. Notably, Lu01-M exhibited significant antiproliferative activity in prostate cancer cell lines (PC3, DU145, and LNCaP). It induced cytotoxicity through multiple mechanisms, including apoptosis, necrosis, autophagy, endoplasmic reticulum stress, and inhibition of colony formation and cell migration. Additionally, Lu01-M also caused G2/M phase cell cycle arrest and DNA damage.

**Table 5:** Compounds with antitumor activity against prostate cancer from actinomycetes secondary metabolites.

Name/compounds	Organism	Cell line	Cytotoxicity	References
EtOAc-Ex	*Streptomyces* sp. KS20	PC-3	IC50: 121.12 μg/mL	Chakraborty et al. (2023)
Rishirilide A	*S. xanthophaeus*	PC-3	IC50: 15.67 μM	Zhang et al. (2022)
Extract	*Streptomyces clavuligerus*	PC-3	GI50: 235.23 μg/mL	Kurt-Kızıldoğan et al. (2022)
4H-Chromen-4-one derivative	*Streptomyces ovatisporus* S4702T	LNCAP	EI50: 9.93 μg mL^−1^	Kurt-Kızıldoğan et al. (2022)
4H-Chromen-4-one derivative	*Streptomyces ovatisporus* S4702T	CaCo-2	EI50: 9.68 μg mL^−1^	Kurt-Kızıldoğan et al. (2022)
A furan-type compound	*Streptomyces* sp. VN1	2276B1	IC50: 157.2 μM	Nguyen et al. (2020)

The EtOAc-Ex (ethyl acetate extract) from *Streptomyces* sp. KS20 showed inhibition of proliferation in A549 and PC-3 cell lines. As the concentration of EtOAc-Ex increased from 12.5 μg/mL to 200 μg/mL, cell viability continuously decreased, with IC50 values of 94.73 μg/mL for A549 cells and 121.12 μg/mL for PC-3 cells. However, the active components responsible for the antitumor activity need further investigation (Chakraborty et al., 2023).

Furthermore, Kurt-Kızıldoğan et al. (2022) isolated a novel 4H-chromene-4-one derivative from marine-derived *Streptomyces* sp. S4702T, with EC50 values of 9.68 μg/mL for CaCO-2 (colorectal cancer cells) and 9.93 μg/mL for LNCaP (prostate cancer cells), while the EC50 for HEK293 (normal cells) was 25.5 μg/mL. This indicates that the 4H-chromene-4-one derivative has lower cytotoxicity in normal cells compared to cancer cells.

### Antitumor activity against other cancers

**Figure 7:**
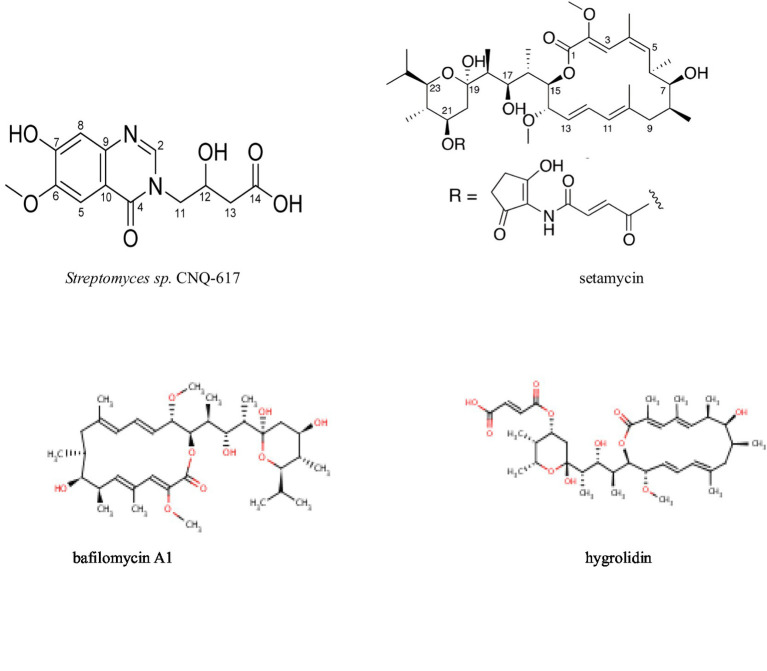
Compounds with antitumor activity against other cancers from actinomycetes secondary metabolites.

Research has shown that secondary metabolites from actinomycetes have potential antitumor activity against various cancers, making them promising candidates for new anticancer drugs (Table 6 and Figure 7). Pulat et al. (2023) isolated a new quinazolinone derivative, actinoquinazolinone, from the fermentation broth of *Streptomyces* sp. CNQ-617 using HPLC-UV detection. At a concentration of 5 μM, actinoquinazolinone inhibited the invasion capability of AGS cells by suppressing epithelial-mesenchymal transition (EMT) markers. Similarly, Zhang et al. (2022) isolated six natural products, including lichirubin A and lupanine A, from wild-type *Streptomyces aureofaciens* No. 2 and its DsxrX mutant. These compounds exhibited anticancer activity against A54, PC-3, and HeLa cell lines. Moreover, Dan et al. (2020) used whole-genome analysis and Global Natural Products Social (GNPS) molecular networking to identify urdamycin E and urdamycin V from *Streptomyces OA293*. These substances showed activity against breast and cervical cancer. Further mechanistic studies demonstrated that urdamycin E exerts its anticancer effect by inhibiting the phosphorylation of mTORC1 and mTORC2 at Ser 2,448 and Ser 2,481, respectively, leading to the inactivation of the mTOR complexes.

**Table 6:** Compounds with antitumor activity against other cancers from actinomycetes secondary metabolites.

Tumor type	Name/Compounds	Organism	Cell line	Cytotoxicity	References
Gastric cancer	*Streptomyces* sp. CNQ-617	Actinoquinazolinone	A549, AGS, and Caco-2	1.5ug/ml	Pulat et al. (2023)
Cervical cancer	Setamycin	*Streptomyces monomycini* RVE129	HeLa	IC50: 24.30 μg/mL	Elias et al. (2022)
Cervical cancer	Extract	*Streptomyces clavuligerus*	Hop-62	GI50: 350.14 μg/mL	Saini et al. (2024)
Fibrosarcoma	Crude methanolic extract of pigment	*Streptomyces* sp.	HT-1080	IC50: 202.13 μg/mL	
Cervical cancer	Crude methanolic extract of pigment	*Streptomyces* sp.	HeLa	IC50: 253.86 μg/mL	
Ovarian cancer	Crude extract	*Streptomyces* sp. strain DSD016T	A2780	Have antiproliferative activity at 2 mg/mL	Sabido et al. (2021)
Gastric cancer	Tsukubaricin	*Streptomyces tsukubaensis*	MGC8031	IC50: 30.34 nM	Wu et al. (2021)
Gastric cancer	Piceamycin	*Streptomyces* sp. SD53 strain	A549	IC50: 0.38 μM	Shin et al. (2020)
Gastric cancer	Bombyxamycin C	*Streptomyces* sp. SD53 strain	A549	IC50: 0.85 μM	Shin et al. (2020)
Leukaemia	Piceamycin	*Streptomyces* sp. SD53 strain	K562	IC50: 0.74 μM	Shin et al. (2020)
Leukaemia	Bombyxamycin C	*Streptomyces* sp. SD53 strain	K562	IC50: 0.96 μM	Shin et al. (2020)
Cervical cancer	Methanolic extract (the main component is C17H29NO14)	*Streptomyces sennicomposti* GMY01	HeLa	IC50: 27.31 μg/m L	Widada et al. (2023)
Pancreatic cancer	Pigment	*Streptomyces tunisiensis* W4MT573222	PAN1	IC50: 1 mg/mL	Ibrahim et al. (2023)
Pancreatic cancer	Cervinomycin C1	*Streptomyces* sp. CPCC 204980	BxPC-3	IC50: 504.7 nM	Hu et al. (2020)
Pancreatic cancer	Cervinomycin C2	*Streptomyces* sp. CPCC 204980	BxPC-3	IC50: 345.4 nM	Hu et al. (2020)
Pancreatic cancer	Cervinomycin C3	*Streptomyces* sp. CPCC 204980	BxPC-3	IC: 754.7 nM	Hu et al. (2020)
Pancreatic cancer	Cervinomycin C4	*Streptomyces* sp. CPCC 204980	BxPC-3	IC50: 801.0 nM	Hu et al. (2020)
Human gastric adenocarcinoma	A furan-type compound	*Streptomyces* sp. VN1	AGS	IC50: 40.5 μM	Nguyen et al. (2020)
Melanoma	A furan-type compound	*Streptomyces* sp. VN1	A375SM	IC50: 84.67 μM	Nguyen et al. (2020)
Glioblastoma	A furan-type compound	*Streptomyces* sp. VN1	U87MG	IC50: 50 μM	Nguyen et al. (2020)
Cervical cancer	Rishirilide A	*S. xanthophaeus*	Hela	IC50: 10.4 μM	Zhang et al. (2022)
Cervical cancer	Lupinacidin A	*S. xanthophaeus*	Hela	IC50: 8.92 μM	Zhang et al. (2022)
Human gastric adenocarcinoma	Oligomycin B	*Streptomyces* sp. FXY-T5	AGS	IC50: 2.67	Feng et al. (2024)
Leukaemia	New zincophorin analogue	NEAU-wh-3-1	K562	IC50: 88.8 μg/mL	
Leukaemia	BE-52211 D	NEAU-wh-3-1	K562	IC50: 11.42 μg/mL	
Leukaemia	1-methoxy-3-methyl-8-hydroxy-anthraquinone	*Amycolatopsis thermoflava* SFMA-103	Lymphoblastic leukaemia cells	IC50: 16.98 μM	
Melanoma	Pradimicin-IRD	*Amycolatopsis* sp. IRD-009	MM 200	IC50: 2.7 μM	
Cervical cancer	Albofungins A	*Streptomyces chrestomyceticus*	Hela	IC50: 0.003 μM	She et al. (2021)
Cervical cancer	Albofungins B	*Streptomyces chrestomyceticus*	Hela	IC50: 0.016 μM	She et al. (2021)
Cervical cancer	Bafilomycin A1	The rare actinobacterium *Catenulispora*	Hela	IC50: 13.0 μM	
Cervical cancer	Hygrolidin	sp. KCB13F192	Hela	IC50: 11.8 μM	Son et al. (2020)
Cervical cancer	Urdamycin E	*Streptomyces* sp. OA293	SiHaL	IC50: 6.46 μg/mL	Dan et al. (2020)
Cervical cancer	Urdamycin V	*Streptomyces* sp. OA293	SiHaL	IC50: 10.39 μg/mL	Dan et al. (2020)

## Antitumor activity of crude extracts-potential compound resources

In the previous sections, we introduced numerous compounds with antitumor activity. However, some studies focus on the anticancer activity of crude extracts, which serve as initial screening experiments. Although these studies do not involve the isolation of active compounds, they still hold significant value in the search for anticancer substances.

For instance, Leonardo screened secondary metabolites from 72 psychrophilic actinomycetes and identified 17 strains with potential antitumor compound production. Bioassay-guided experiments showed that crude extracts from Streptomyces exhibited significant antiproliferative activity. These natural products inhibited the proliferation of breast cancer cells (MCF-7), glioblastoma (U251), lung/non-small cell lung cancer (NCI-H460), and renal cancer cells (786-0) (Silva et al., 2020).

Similarly, Li et al. tested the bioactivity of crude extracts from *Actinocorallia populi* A251T, demonstrating anticancer and antidiabetic activity against the HepG2 tumor cell line, although active compound isolation was not conducted. Moreover, Wang et al. screened actinomycete strains from the rhizosphere soil of wheat and isolated 39 strains, including some from Hebei, China. Among these, extracts from 14 strains inhibited the growth of the colon cancer cell line HCT-116.

In addition, Saini et al. (2024) found that organic extracts from Streptomyces exhibited low GI50 values against MCF-7, Hop-62, SIHA, and PC-3 cells, showing strong growth inhibitory effects on human breast and prostate cancer cells. Subsequent HR-LC MS analysis identified various secondary metabolites from the organic and aqueous extracts, including epirubicin (Epi).

Taken together, these findings suggest that crude extracts possess strong potential for the development of antitumor substances. Purification and further study of these crude components could contribute to the development of new anticancer drugs.

### Development and utilization of secondary metabolites from actinomycetes: OSMAC strategy

The OSMAC (One Strain Many Compounds) strategy, proposed by Zeeck et al. in 2002, has become an effective method for activating microbial silent biosynthetic gene clusters (BGCs) and discovering novel natural products (Romano et al., 2018). Unlike traditional genetic editing or chemical induction methods, the OSMAC strategy activates silent BGCs by altering microbial cultivation conditions, such as media composition, physical conditions, chemical additives, and co-cultivation. This approach allows the production of new secondary metabolites in microbes without genetic manipulation. The application of this strategy has not only enhanced the diversity of microbial metabolic products but also provided new avenues for drug discovery, especially in the field of actinobacteria. By adjusting the external environment, the OSMAC strategy can trigger the latent metabolic potential of microbes, greatly increasing chemical diversity and the discovery of bioactive compounds (Romano et al., 2018).

In recent years, significant progress has been made in the study of actinobacterial secondary metabolites based on the OSMAC strategy. For example, Li Y. et al. (2023) fermented three actinobacteria strains in four different media, successfully activating multiple silent gene clusters and obtaining 12 new secondary metabolites. These studies demonstrate that by carefully selecting the culture media and fermentation conditions, the OSMAC strategy can effectively activate potential metabolic products in actinobacteria and discover new compounds. In another study of marine actinobacterium YB104 (Carroll et al., 2020), researchers altered media components and fermentation conditions, successfully isolating 17 new compounds, many of which exhibited antimicrobial activity. Similarly, OSMAC has been applied to *Saccharothrix fischeri*, *Streptomyces* sp. *SCSIO 15079*, and other strains, resulting in the discovery of novel natural products with significant pharmacological potential (Li Y. et al., 2023; Romano et al., 2018; Carroll et al., 2020). Through this method, the diversity of metabolic products in actinobacteria has been greatly enriched, providing valuable chemical resources for drug development.

However, despite the significant achievements of the OSMAC strategy in the study of actinobacterial secondary metabolites, there are still some challenges. First, although this strategy does not rely on genetic manipulation, its effectiveness depends on fine-tuning various cultivation conditions, which may require extensive screening and optimization. Moreover, microbial responses to cultivation conditions can vary, making it complex and time-consuming to identify the optimal conditions for activating silent gene clusters. Additionally, the synthesis of some secondary metabolites may be strongly influenced by ecological factors, making it difficult to replicate natural environments in laboratory settings. Nevertheless, with the development of genomics and metabolomics, the combination of the OSMAC strategy with modern biotechnology (such as high-throughput screening and metabolic engineering) has greatly increased the efficiency of discovering microbial metabolites. In the future, combining the OSMAC strategy with gene editing, metabolomics, and other techniques will provide more possibilities for actinobacterial secondary metabolite research and accelerate the discovery and development of new drugs.

### Co-cultivation and mixed cultivation strategies

Co-cultivation strategy, as an important microbial engineering technique, has demonstrated tremendous potential in the production of secondary metabolites in recent years. Similar to how intercropping and mixed cropping in agriculture increase crop yields, improve soil health, and promote biodiversity, co-culturing microorganisms can significantly enhance the diversity and yield of their metabolic products. By simulating the symbiotic and competitive relationships between microorganisms in natural environments, co-cultivation can activate silent gene clusters, discover and produce new secondary metabolites, and increase the yield of known metabolites.

Similar to how intercropping different crops can promote mutual growth and suppress pests, the interactions between microorganisms in co-cultivation can trigger the metabolic potential of different microbes, resulting in the production of a series of new bioactive compounds. For example, during the co-cultivation of *Streptomyces* sp. and *L. minima*, researchers discovered a new secondary metabolite—ulleungdolin—with a significant increase in yield (Arora et al., 2020). This illustrates that the interactions between microorganisms notably enhance the diversity of their metabolic products. This phenomenon mirrors the mutually beneficial relationships seen in intercropping, which significantly improve biodiversity in ecosystems.

Co-cultivation not only activates silent genes but also significantly increases the yield of known secondary metabolites through the synergistic interactions between microorganisms. For instance, Arora et al. (2020) discovered a new secondary metabolite, penicinoline, by co-culturing the marine fungus *Penicillium* sp. and the bacterium *Pseudomonas aeruginosa*, and the yield of this compound was significantly enhanced under co-cultivation conditions (Zhang J. et al., 2021). Similar to the competition and cooperation between crops in mixed cropping, the microorganisms in co-cultivation also promote the generation of products through resource allocation, metabolic exchange, and other mechanisms. In agriculture, mixed cropping often enhances soil fertility and improves crop resistance to stress by promoting the interactions between crops; similarly, in microbial co-cultivation, these interactions promote metabolic activities to increase yield.

Despite the significant progress co-cultivation strategies have made in enhancing secondary metabolite yield, several challenges remain. For instance, the competitive relationships in co-cultivation conditions may lead to the suppression of certain species, or antagonistic effects on key metabolic pathways. These issues require more in-depth research to optimize the design and conditions of co-cultivation systems. Additionally, the efficient screening of co-culture combinations and the precise control of metabolite synthesis are still technical bottlenecks. Future research could draw on the success of mixed cropping in agriculture, combining high-throughput screening and genome-editing technologies to further enhance the application potential of co-cultivation strategies.

In conclusion, microbial co-cultivation strategies show great potential in improving secondary metabolite yields and increasing the diversity of metabolites, much like intercropping in agriculture. With further in-depth mechanistic research and technological innovation, this strategy is expected to play an increasingly important role in the development of natural products and the microbial fermentation industry in the future.

### Other chemical methods

Diversifying culture conditions, ribosomal engineering, and other chemical methods are commonly used in the cultivation of actinomycetes. Researchers have activated the cryptic BGC of chattamycin in *Streptomyces chattanoogensis* L10 by overexpressing regulatory genes and using ribosomal engineering. Another study altered culture conditions by growing *Streptomyces UKAQ23* under aerobic conditions with assimilable carbon and nitrogen sources in solid-state fermentation. This resulted in the production of actinomycins X2 and D, which are antibiotics use.

Additionally, sensitizing microbial cells with external inducers is a recognized strategy. Using minimally effective concentrations of small organic molecules can induce silent BGCs in actinomycetes cultures to produce secondary metabolites. Liu et al. (2022) combined promoter engineering with optimized fermentation conditions to activate a cryptic aromatic polyketide BGC, identifying three new pentangular polyphenolic compounds, Lanthomicin A–C. Lanthomicin A, in particular, exhibited antiproliferative activity against the lung cancer cell line A-549, with an IC50 of 0.17 μM, highlighting its potential as a drug.

These methods chemically induce the activation of silent BGCs in actinomycetes, leading to the production of secondary metabolites. The widespread application of these approaches has provided numerous new bioactive secondary metabolites in recent years (Diarra et al., 2024).

### Potential resources of marine-derived actinomycetes

In recent decades, the ocean has been proven to be an important repository of rare actinomycetes.

Marine-derived actinomycetes have complex relationships with unique marine environments, such as those with high salinity and specific aquatic organisms. These interactions lead to unique genetic characteristics and interesting secondary metabolic pathways, making marine actinomycetes a potential target for mining rare, small molecular resources (Chen et al., 2021; Jagannathan et al., 2021). Therefore, marine actinomycetes are considered to be underutilized natural product libraries, which can provide a variety of compounds with unique structures and biological activity.

Bahrami reviewed 232 chemical structures with anti-colorectal cancer activity from actinomycetes, including quinones, lactones, alkaloids, peptides, and glycosides. Most of these natural products came from marine actinomycetes (79.02%), followed by terrestrial and endophytic actinomycetes, showing the potential value of marine-derived actinomycetes (Chen et al., 2021; Hafez Ghoran et al., 2022). It is reported that between 2007 and 2017, marine actinomycetes produced a total of 267 new compounds (Subramani and Sipkema, 2019), many of which have antibacterial, anticancer, antiviral and anti-inflammatory activities. For example, Salinosporamide A isolated from marine actinomycetes, is a compound with strong anticancer activity that is currently in the stage of clinical research.

## Challenges in the development of actinomycete metabolites

### Re-discovery of secondary metabolites

Despite the average discovery of 1,000 new natural products annually over the past two decades, the frequent re-discovery of known compounds remains a significant bottleneck in this field (Diarra et al., 2024). This redundancy not only wastes research resources but also slows down the discovery of new compounds. To overcome this issue, scientists are exploring new technologies and strategies, such as high-throughput screening, metabolomics, and genome mining, to improve the rate of new compound discoveries.

### Production and purification challenges

The production and purification of secondary metabolites from actinomycetes pose significant technical challenges. Secondary metabolites are often present in low concentrations within actinomycete cells, necessitating large amounts of biomass to extract sufficient active substances. Besides the issue of low yield, the complex structures of these compounds further complicate the purification process. Additionally, the yield and variety of secondary metabolites can vary significantly under different cultivation conditions, adding to the complexity of the production process.

For example, changing the composition of the medium, temperature, and pH can all affect the yield and variety of metabolites. Therefore, the production process requires detailed optimization to ensure high yield and purity of the target compounds.

### Toxicity and side effects

Many secondary metabolites from actinomycetes exhibit significant biological activity, which makes them attractive targets for drug development. However, this also brings potential toxicity and side effects. During drug development, it is crucial to conduct thorough toxicity evaluations of these compounds to ensure their safety and efficacy. This often requires extensive *in vitro* and *in vivo* testing, increasing development costs and time. Some existing drugs derived from actinomycetes also face toxicity and safety issues. For instance, actinomycin D, an antitumor drug, has limited use in various cancers due to its toxic effects, including tissue necrosis, bone marrow suppression, skin toxicity, and gastrointestinal toxicity. Similarly, despite being a potent anticancer drug, mitomycin C’s clinical application is severely hindered by its toxicity to bone marrow and other tissues (Gavriilidou et al., 2022).

## Prospects for the development of secondary metabolites from actinomycetes

### Genome mining

In recent years, with the advancement of genomics and metabolic engineering, genome sequencing has been widely applied in scientific research. The latest progress in the genome sequencing of actinomycetes has revealed a significant “innovation gap” between biosynthetic potential and isolated bioactive secondary metabolites (SMs). The genomes of these actinomycetes typically contain many biosynthetic gene clusters (BGCs) that encode pathways for producing SMs, but a large number of these gene clusters remain silent, encoding molecules that have not yet been observed (Diarra et al., 2024).

Exploring these silent genes holds the potential to uncover rare molecules that are otherwise difficult to obtain. Gavriilidou et al. (2022) used two different algorithms to mine sequencing data from sediment bacteria to identify BGCs and group them according to the chemical families of the encoded compounds. They identified many unexplored taxa with significant biosynthetic potential. Remarkably, the authors estimated that only 3% of the potential natural products encoded in bacterial genomes have been experimentally characterized (Ngamcharungchit et al., 2023b).

Research using the AntiSMASH tool analyzed the metagenomic sequencing results of *Nonomuraea corallina* sp., discovering BGCs encoding many metabolites. These compounds, including several anticancer compounds, had not been previously reported from Nonomuraea species, suggesting the discovery of new targets (Abdelaleem et al., 2023).

Zhang et al. (2022) identified a new lichenysin biosynthetic gene cluster from *Streptomyces aureofaciens* No. 2. This cluster contained a rare cluster-specific phosphopantetheinyl transferase (PPTase), confirmed through genetic mutation and biochemical characterization. Liu et al. (2022) activated a cryptic aromatic polyketide BGC and identified a new pentangular polyphenolic compound, Lanthomicin A, which exhibited anticancer activity against lung cancer.

These studies encourage scientists to explore various technologies and strategies to de-silence these BGCs, trigger their expression, and identify new natural products and molecules.

### Metabolomics techniques

Metabolomics is considered a highly valuable tool for analyzing natural products under specific conditions. This technology aims to quickly identify known metabolites in samples through gas or liquid chromatography combined with high-resolution electrospray ionization mass spectrometry (HRESIMS) or nuclear magnetic resonance (NMR) spectroscopy. It has been widely applied in the study of secondary metabolites from actinomycetes (Salehghamari et al., 2022; Hwang et al., 2022).

Abdelaleem’s team utilized metabolomics tools and statistical analysis, using MetaboAnalyst 4.0 to screen and dereplicate known metabolites in extracts from 12 sponge-associated actinomycetes, distinguishing between the extracts and identifying numerous secondary metabolites. Sekurova et al. (2022) used transcriptomics and metabolomics to study the significant changes in gene expression profiles and their different effects on secondary metabolite biosynthesis and central metabolic pathways induced by ethanol shock. This research helps to understand the complex mechanisms behind environmental factor-induced regulation of secondary metabolite biosynthesis, providing new insights for the development of secondary metabolites from actinomycetes (Ashraf et al., 2022). Additionally, many studies have relied on mass spectrometry and metabolomics to discover new secondary metabolites of research interest (Balsamo et al., 2022).

Metabolomics can be used not only to isolate and explore new secondary metabolites from strains but also to identify promising anticancer natural products. Joseph’s team established an integrated system based on metabolomics, combining single-cell metabolomics with an immunogenic cell injury module. This integration allows detailed analysis of cellular responses to natural products at the single-cell level, aiding in the discovery of effective compounds. Anglada-Girotto et al. (2022) generated a reference map of metabolic changes from CRISPR interference of 352 genes across all major basic biological processes. By comparing gene-related maps with metabolic changes induced by 1,342 drugs, they performed *de novo* functional predictions of compounds, revealing drugs with non-traditional modes of action and providing new avenues for the development of anticancer drugs derived from actinomycetes (Moon et al., 2019).

The development of new technologies, especially new “omics” methods, which are extremely effective and sensitive for studying secondary metabolites, combined with genome mining, should aid in the development of new emerging bioactive compounds. This has the potential to have a profound impact on human health, particularly in the field of cancer prevention and treatment.

### Introduction of the HiTES method

The HiTES (High-Throughput Elicitor Screening) method is designed to discover small molecule inducers of silent biosynthetic gene clusters (BGCs). By utilizing chemical-genetic screening, this method aims to identify small molecule inducers that can activate these silent gene clusters. This targeted activation of BGCs in genetically tractable strains can significantly enhance the biosynthesis of secondary metabolites (SMs). The fundamental principle involves introducing a reporter gene into the target silent BGC to provide a quick expression readout.

The specific steps are as follows:

Introduction of the reporter gene: A reporter gene is introduced into the target BGC so that when the BGC is activated, the reporter gene expresses an easily detectable marker (such as a fluorescent protein).
Chemical library screening: Various small molecules from a chemical library are used to treat the strain containing the reporter gene to observe which molecules can activate the reporter gene.
Result analysis: By detecting the expression of the reporter gene, small molecules that can induce BGC expression are identified, and their induction mechanisms are further studied.The HiTES method has been successfully applied to various bacteria, including the *Gram-negative bacterium Burkholderia thailandensis*. This method allows targeted activation of BGCs in genetically tractable strains, significantly enhancing SM biosynthesis. Some specific application cases include:
Moon et al. (2019) applied this strategy to *Saccharopolyspora cebuensis* and identified a new thiopeptide, cebulantin. Subsequent bioactivity assays revealed that it is a selective antibiotic against *Gram-negative bacteria*, particularly Vibrio species. Lee et al. (2024) used Bioactivity-HiTES on the oral bacterium *Actinomyces matruchotii*, screening 3,120 clinical drugs as potential elicitors and identifying two hidden metabolites, methylated indole-3-acetic acid (MIAA) and indole-3-acetic acid (IAA) (Li et al., 2022). Li targeted *Streptomyces rimosus* using MALDI-MS-guided HiTES, mapping the total secondary metabolome of *S. rimosus* and identifying three cryptic featured products (Sajid et al., 2021).

With the advancement of genomic sequencing technologies, increasing amounts of microbial genome data have revealed the vast biosynthetic potential of inactive secondary metabolites. Actinobacteria, known for producing a wide range of secondary metabolites, are widely used in drug development, particularly in the fields of antibiotics, anti-cancer agents, and immunomodulators. As HiTES technology evolves towards single-cell transcriptomics and dynamic monitoring, it offers the potential to more precisely analyze the gene expression patterns of actinobacteria during different growth stages, environmental stimuli, and genetic regulation, particularly those genes associated with secondary metabolite biosynthesis. In the future, HiTES is expected to integrate AI and machine learning technologies to automatically identify key genes and regulatory networks related to secondary metabolite production within vast transcriptomic datasets, enhancing the understanding of biosynthetic pathways. Through real-time monitoring and cross-species comparisons, HiTES could also help uncover metabolic product variations in different actinobacteria under diverse conditions, facilitating the discovery and optimization of novel drugs. Furthermore, with improved sensitivity and reduced costs, HiTES is poised to find widespread application in the screening, optimization, and engineering of actinobacteria strains for secondary metabolite production, accelerating the development of natural products.

### Synthetic biology

It is well known that post-synthetic modifications such as methylation, glycosylation, phosphorylation, and alkylation can affect pharmacokinetics (PK) and pharmacodynamics (PD). These modifications have also been applied to the development and improvement of drugs derived from actinomycetes (Mohan et al., 2022). For example, actinomycin D is approved for treating certain cancers, but its side effects, such as tissue necrosis, bone marrow suppression, skin toxicity, and gastrointestinal toxicity, pose significant challenges. Similarly, doxorubicin has dose-limiting bone marrow suppression, lethal cardiotoxicity, and cytotoxicity to normal cells. To improve the solubility of drugs in water and overcome the adverse effects of chemotherapeutic agents, researchers have developed synthetic or semi-synthetic methods to create newer derivatives. For instance, the clinical development of rapamycin as an anticancer drug was initially halted due to its poor hydrophilicity. Subsequently, its water-soluble derivatives (everolimus and temsirolimus) were found to be effective antitumor agents and were approved for treating renal cancer, pancreatic neuroendocrine tumors, and subependymal giant cell astrocytoma (Chen et al., 2020). These examples highlight the importance of using natural compounds as template structures to create semi-synthetic derivatives with chemical novelty, bioavailability, solubility, and lower physiological toxicity.

In addition to modifying compounds, direct intervention in the biosynthetic pathways of secondary metabolites can enhance their production. N-Acetylglucosamine (GlcNAc) is a key signaling molecule that controls the development and antibiotic synthesis in Streptomyces. Under chemically defined conditions, GlcNAc can significantly increase the production of bleomycin. Based on this, researchers constructed a co-expressing strain OBlmT/manab with blmT, mana, and manB genes. Through GlcNAc regulation and auxiliary metabolic profile analysis, the yields of bleomycin A2 and B2 were ultimately increased to 61.79 and 36.9 mg/L (Carroll et al., 2020), respectively. Intervening in the biosynthetic pathways of secondary metabolites provides new avenues for developing secondary metabolites from actinomycetes.
