# Correction: Biological activity of secondary metabolites of actinomycetes and their potential sources as antineoplastic drugs: a review

**DOI:** 10.3389/fmicb.2025.1672871

**Published:** 2025-08-08

**Authors:** Sun Rui, Guo Fengrui, Zhang Yining, Shao Hong, Yang Xuewen, Wang Changping, Yang Chunjia

**Affiliations:** ^1^College of Biology and Agriculture, Jiamusi University, Jiamusi, China; ^2^School of Basic Medicine, Jiamusi University, Jiamusi, China

**Keywords:** actinomycetes, antitumor, cancer, secondary metabolite, activity

An incorrect number was provided for Heilongjiang Province. The correct number is (2021-KYYWF-0574).

The correct funding statement reads:

The author(s) declare that financial support was received for the research and/or publication of this article. This study was funded by the Basic Scientific Research Business Expenses of Colleges and Universities in Heilongjiang Province (2021-KYYWF-0574).

The original version of this article has been updated.

